# Micronutrient gaps during the complementary feeding period in South Asia: A Comprehensive Nutrient Gap Assessment

**DOI:** 10.1093/nutrit/nuaa144

**Published:** 2021-03-08

**Authors:** Ty Beal, Jessica M White, Joanne E Arsenault, Harriet Okronipa, Guy-Marino Hinnouho, Zivai Murira, Harriet Torlesse, Aashima Garg

**Affiliations:** 1 Global Alliance for Improved Nutrition, Washington, DC, USA; 2 Department of Environmental Science and Policy, University of California, Davis, Davis, California, USA; 3 United Nations Children's Fund (UNICEF), New York, New York, USA; 4 Institute for Global Nutrition, University of California, Davis, Davis, California, USA; 5 Intake, Center for Dietary Assessment, FHI Solutions, Washington, DC, USA; 6 Department of Population Medicine and Diagnostic Sciences, Cornell University, Ithaca, New York, USA; 7 Helen Keller International, New York, New York, USA; 8 UNICEF, Regional Office for South Asia, Kathmandu, Nepal

**Keywords:** CONGA, micronutrient deficiencies, nutrient adequacy, nutrient gap assessment, South Asia

## Abstract

Micronutrient malnutrition is a key driver of morbidity and mortality for millions of children in South Asia. Understanding the specific micronutrients lacking in the diet during the complementary feeding period is essential for addressing undernutrition caused by inadequate diets. A Comprehensive Nutrient Gap Assessment was used to synthesize diverse evidence and estimate the public health significance of complementary-feeding micronutrient gaps and identify evidence gaps in 8 countries in South Asia. There were important gaps across the region in iron, zinc, vitamin A, folate, vitamin B_12_, and, to a lesser extent, calcium and vitamin C. The most nutrient-dense, whole-food sources of these micronutrients include liver, small fish, eggs, ruminant meat, and dark leafy greens. Investment is needed in some countries to collect data on micronutrient biomarkers and dietary intakes. A food systems approach is essential for improving child diets and reducing malnutrition, which affects millions of children, their futures, and society at large across South Asia and beyond.

## INTRODUCTION

A quarter of the global population of children younger than 5 years live in South Asia (*n* = 170 million children),^1^ a third of whom are stunted (*n* = 56 million) and 15% of whom are wasted (*n* = 25 million).^2^ Deficiencies of essential vitamins and minerals are widespread among children in the region.^3^ For example, in India, in which more than two-thirds of South Asia’s children younger than 5 years (*n* = 117 million) live, the following micronutrient deficiencies among children aged 12–59 months are widespread: iron (32%), folate (23%), zinc (19%), vitamin A (18%), vitamin B_12_ (14%), and vitamin D (14%).^4^ The consequences of this high burden of undernutrition among children are severe, including poor cognitive development, failure to reach their full potential, and death.^5^^,^^6^

One of the main causes of undernutrition in the region is poor diet.^3^^,^^7^ Only 20% of children aged 6–23 months in South Asia consume the minimum recommended number of food groups each day.^8^ Consumption of animal-source foods, in particular, is low.^9^ Improving diets of young children is an important component of efforts to achieve the World Health Assembly and Sustainable Development Goals nutrition targets. Identification of micronutrient and dietary gaps during the complementary feeding period is essential to inform policies and programs designed to improve child health and nutrition.^10^

Existing evidence that could be used to inform the understanding of micronutrient gaps in South Asia, including prevalence of micronutrient deficiencies or inadequate intakes, comes from disparate data sources of varying quality, representativeness, and recency. Moreover, this evidence, to our knowledge, has not been synthesized to produce a comprehensive and clear picture of the magnitude and significance of micronutrient gaps across countries in South Asia. Without this understanding, it is difficult to identify the public health significance of micronutrient gaps in diets of young children and how to best address them.

In another article in this *Nutrition Reviews* supplement, Beal et al^6^ describe a method of compiling available evidence from a variety of sources to assess the public health significance of nutrient gaps and identify evidence gaps. This approach, called Comprehensive Nutrient Gap Assessment (CONGA), was used to assess the micronutrient gaps in young children’s diets in the 8 countries of South Asia. After identifying micronutrient gaps, the most nutrient-dense, whole-food sources (ie, those with minimal processing and typically only 1 or 2 ingredients) of these micronutrients that are available in the region were identified.

## METHODS

To identify nutrient gaps—shortfalls in the diet that lead to inadequate nutrient intakes—the analysis followed the 8-step method for conducting a CONGA, described in detail by Beal et al^6^ in this journal supplement. The target population was children 6–23 months of age in the 8 countries of South Asia: India, Pakistan, Bangladesh, Afghanistan, Nepal, Sri Lanka, Bhutan, and the Maldives. Micronutrients assessed were those identified in the literature as commonly lacking in the diets of infants and young children during the complementary feeding period: iron, vitamin A, zinc, calcium, iodine, thiamine, niacin, vitamin B_12_, vitamin B_6_, folate, and vitamin C.^11^ Evidence sources were identified (CONGA step 1) by (1) searching relevant global databases, including Demographic and Health Surveys (DHS) and DHS STATcompiler (dhsprogram.com), Multiple Indicator Cluster Surveys (mics.unicef.org), and United Nations Children’s Fund (UNICEF) global databases on infant and young child feeding, malnutrition, and iodine (data.unicef.org); (2) requesting relevant resources from UNICEF Regional Office South Asia and UNICEF and Global Alliance for Improved Nutrition country offices; and (3) conducting keyword searches in Google and Google Scholar. Keywords (Table S1 in the Supporting Information online) on specific evidence types (eg, nutrient intake) were searched in Google and Google Scholar, and keywords for specific report types or gray literature (eg, national micronutrient survey) were searched solely in Google.

Studies were included if they contained information on any of the 5 evidence types relevant for assessing nutrient gaps outlined in the CONGA methods paper:^6^: (1) biological, clinical, and functional markers, (2) nutrient adequacy of individual diets, (3) nutrient adequacy of household diets, (4) nutrient adequacy of national food supplies, and (5) nutrient-informative food group intake of individuals or households (eg, iron-rich foods). Priority was given to evidence on children aged 6–23 months, but evidence was also included on populations inclusive of young children (eg, children younger than 5 years) and relatively similar age and sex groups (eg, children aged 36–59 months). For micronutrient markers that do not vary substantially across age groups, such as urinary iodine concentration, data on older age groups were included. Studies on micronutrient supplementation coverage and Cost of the Diet analyses that could inform micronutrient gap assessment were also included. Studies with sample sizes of < 50 participants , geographic representation of < 10% of the national population, highly vulnerable participants, and those in which data collection ended before the year 2000 were excluded. Table S2 available in the Supporting Information online contains a spreadsheet with information from all included evidence sources documented according to the CONGA method (CONGA step 1).^6^

Two experts (T.B. and J.M.W.) conducted CONGA steps 2–6 to determine ratings for all 11 micronutrients in each of the 8 countries. In step 2, data points in the country-specific spreadsheets were reviewed and assigned an implied nutrient gap burden score per the CONGA methodology.^6^ Weights were then assigned to the metadata to calculate an overall evidence weight score for each data point in step 3. Assigning an evidence weight score on the basis of metadata helped account for the fact that not all data points are equally valuable or robust. Weight scores were systematically applied to the metadata for each data point (see Beal et al^6^), ensuring that the most recent, representative, and relevant data were weighted more heavily when assessing nutrient gaps.

In step 4, the implied nutrient gap burden scores and evidence weight scores were used to calculate a quantitatively derived nutrient gap burden rating. The quantitative burden rating was calculated using data only from the aforementioned 5 core evidence types (excluding “other” data) that were collected in 2010 or later and that were for age or sex groups similar to children 6–23 months of age. The data points excluded from this calculation were considered in step 5. A numeric score was calculated for each nutrient in each country, using the weighted mean of the implied nutrient gap burden score (where the evidence weights are the weight scores) and assigned a label of high, moderate, low, or negligible, per CONGA methodology.^6^

In step 5, the quantitative nutrient gap burden scores were reviewed alongside the totality of evidence for each nutrient, including data points that did not meet criteria for inclusion in the quantitative burden score calculation and additional available information for each data point (eg, temporal trends for data points, where available) to determine whether the final rating assigned to the nutrient gap should deviate from the quantitatively derived rating. A final qualitative rating of high, moderate, low, or negligible was assigned to each nutrient for each country, and any deviation from the quantitative burden score was documented and explained.

The certainty of evidence was rated for each nutrient burden (high, moderate, low, or unknown) in step 6 using established CONGA methodology criteria,^6^ by which the evidence weight scores from step 3 are considered and level of agreement between data points. These criteria-based ratings were also subjected to a final qualitative review, considering all evidence (including data points for which an evidence weight score was not possible), to determine whether the final certainty rating should deviate from the criteria-based rating. Any deviations were discussed and documented.

In step 7, all other coauthors, who are subject matter and contextual knowledge experts, reviewed the final qualitative nutrient gap burden and evidence certainty ratings produced in steps 5 and 6, respectively. Disagreements with final qualitative ratings were discussed and critically re-evaluated. Ratings were finalized only when consensus was achieved and documentation of additional considerations or deviations from quantitative burden scores was added. Table S2 in the Supporting Information online contains a spreadsheet with all data points included in each CONGA, data sources, and nutrient gap and certainty scores.

For identified micronutrient gaps (ie, those that received a nutrient gap burden rating *and* a certainty-of-evidence rating of at least moderate) and potential micronutrient gaps (ie, those that received a nutrient gap burden rating of at least moderate but a certainty-of-evidence rating of low), the most nutritious, regionally available, whole-food sources of these micronutrients were identified on the basis of food-composition data from Bangladesh,^12^ India,^13^ and the US Department of Agriculture,^14^ and consumption patterns from household consumption and expenditure surveys in Bangladesh, India, and Pakistan (as specified by Ryckman et al^15^). With the exception of liver, for which there were insufficient consumption data, foods were eligible for consideration if they were consumed by at least 10% of households nationally (typically over 1 to 2 weeks) in 1 of the 3 sample countries.

Because many whole foods are good sources of several micronutrients, these foods were also assessed for how well they met the needs of 6 micronutrients critical for child growth and development and likely to be lacking in South Asian diets: namely, iron, vitamin A, zinc, folate, vitamin B_12_, and calcium. This metric, called average share of requirements, was calculated as the average proportion of daily requirements from complementary foods for these 6 micronutrients on the basis of a 100-g quantity (each micronutrient capped at 100% of daily requirements). The portion size of each food required to achieve an average of 33.3% of requirements (again, capped at 100% of requirements for each micronutrient)—the equivalent of 100% of requirements for 2 micronutrients or 33.3% of requirements for all 6 micronutrients—was also calculated to demonstrate the ideal foods to fill ≥ 2 important micronutrient gaps simultaneously. Adjustments for differences in bioavailability between plant and animal-source foods were made for iron and zinc. For more details on how micronutrient density of and average share of requirements for identified foods were calculated and how local availability was determined, refer to other articles in this journal supplement.^15^^,^^16^

## EVIDENCE SUMMARY

A total of 321 qualifying data points from 50 evidence sources (see Supporting Information online) were identified in the literature review (CONGA step 1), and 137 of these met criteria for inclusion in the quantitative burden score (CONGA steps 3 and 4) (Table 1). Pakistan (*n* = 63) and India (*n* = 59) had the highest number of data points identified in the literature review, followed by Bangladesh (*n* = 41), Sri Lanka (*n* = 40), Nepal (*n* = 38), Afghanistan (*n* = 31), the Maldives (*n* = 30), and Bhutan (*n* = 19). In several countries, fewer than half of these data points met qualifying criteria for inclusion in the quantitative micronutrient gap burden score (in Bhutan, only 1 data point qualified).

**Table 1:** Number of data points included overall and that qualified for the quantitative burden scores by country

	No. of data points included overall	No. of data points that qualified for quantitative burden scores
India	59	23
Pakistan	63	20
Bangladesh	41	25
Afghanistan	31	18
Nepal	38	19
Sri Lanka	40	19
Bhutan	19	1
Maldives	30	12
Total	321	137

The variety of evidence sources differed by country. Pakistan, for example, had a 2018 national nutrition survey, 2017–2018 DHS, a 2018 Fill the Nutrient Gap report, a 2017 Cost of the Diet analysis, and 2018 Optifood analysis, among others. In contrast, Bhutan had a 2015 national nutrition survey with limited relevant data to extract and few other nutrition-specific evidence sources. Recency of data collection for identified evidence varied across countries; however, all countries had at least 1 evidence type collected within the previous 6–7 years. All countries also had relevant nationally representative data available for several micronutrients and/or evidence types. Relevance to the age group of interest varied by evidence type and data source; however, the majority of data reviewed were for children younger than 5 years or 6–59 months old.

The number of data points varied considerably across micronutrients and evidence types (Table 2). Biochemical or functional markers were identified for 7 of the 11 micronutrients of interest. The most common biochemical or functional markers available were for iron, zinc, and vitamin A. Prevalence of deficiencies in iron, vitamin A, and zinc for children younger than 5 years, 6–59 months old, or 12–59 months old were available in all countries except Bhutan. Estimates of anemia prevalence were available for all countries, but these were not used to estimate iron deficiency, because of limited information on the proportion of anemia due to iron deficiency in these populations. Prevalence of folate deficiency was available for children aged 6–23 months, 6–59 months, or 12–59 months in Pakistan, India, and Nepal, and for other age groups in Afghanistan and Bangladesh. Prevalence of vitamin B_12_ deficiency was available for children aged 6–59 months or 12–59 months in Pakistan and India, and for other age groups in Bangladesh. All countries except Bhutan had at least 1 biochemical or functional marker of iodine deficiency (ie, prevalence of deficiency, median urinary iodine concentration, or total goiter rate); however, most estimates were for school-aged children. Sri Lanka was the only country with an estimate of calcium deficiency in children (6–59 months old), and Pakistan assessed calcium deficiency for women of reproductive age. No qualifying biochemical evidence for any age group was identified in the literature search for niacin, thiamine, vitamin C, or vitamin B_6_—similar to a CONGA in 6 countries in Eastern and Southern Africa.^17^

**Table 2:** Number of data points included overall and that qualified for the quantitative burden scores by evidence type and micronutrient^a^

Evidence type	Iron	Zinc	Vit A	Folate	Vit B_12_	Ca	Vit C	Niacin	Vit B_1_	Iodine	Vit B_6_
Biological and functional markers	8 (7)	7 (6)	9 (5)	5 (4)	3 (2)	2 (1)	0 (0)	0 (0)	0 (0)	11 (8)	0 (0)
Nutrient adequacy of individual diets	0 (0)	0 (0)	0 (0)	0 (0)	0 (0)	0 (0)	0 (0)	0 (0)	0 (0)	0 (0)	0 (0)
Nutrient adequacy of household diets	1 (1)	1 (1)	1 (1)	0 (0)	0 (0)	1 (1)	1 (1)	1 (1)	1 (1)	0 (0)	0 (0)
Nutrient adequacy of national food supplies	7 (7)	13 (7)	13 (7)	12 (7)	13 (7)	13 (7)	7 (7)	7 (7)	11 (7)	0 (0)	7 (7)
Nutrient-informative food-group intake of individuals or households	10 (10)	0 (0)	8 (8)	0 (0)	0 (0)	0 (0)	0 (0)	0 (0)	0 (0)	13 (9)	0 (0)

a The parentheses enclose the number of data points that qualified for the quantitative burden scores. *Abbreviations:* Ca, calcium; Vit, vitamin.

In contrast to the CONGA in Eastern and Southern Africa,^17^ no qualifying evidence on micronutrient adequacy of individual diets was identified in the literature search for any country in South Asia. Some evidence was identified that inferred micronutrient adequacy (eg, the proportion of children who consumed < 70% of the recommended daily allowance) but was not presented in ways that allowed for clear interpretation. Therefore, this evidence was classified as *other* and considered in the qualitative assessment (CONGA step 5). Data on micronutrient adequacy of household diets were identified in Bangladesh only. Data on micronutrient adequacy of national food supplies were identified for all micronutrients considered except iodine, and for all countries except Bhutan. Evidence containing micronutrient-informative food-group intake of individuals or households was available for iron and vitamin A (from consumption of iron- and vitamin A–rich foods in children aged 6–23 months) and iodine (from data on coverage of iodized salt). At least 1 data point classified as other evidence (eg, micronutrient supplementation, Optifood analyses) was identified for all micronutrients and all countries.

## RATINGS ADJUSTMENTS

Adjustments to quantitative burden ratings involved a critical qualitative assessment of the totality of evidence, including evidence excluded from the quantitative burden rating, “other” evidence, and temporal trends. Table S2 in the Supporting Information online includes all initial and final qualitative micronutrient gap burden and certainty-of-evidence ratings. Although most initial ratings remained the same, 15 of 88 micronutrient gap burden ratings and 6 of 88 certainty ratings were adjusted. Among ratings that changed, burden ratings tended to reduce in severity, whereas certainty ratings tended to increase in certainty, but there were some exceptions.

Calcium had the most changes to initial burden ratings (*n* = 4), all of which reduced in severity (*n* = 3 changed from high to moderate, *n* = 1 from moderate to low), because initial ratings were largely based on high estimates of inadequacy in the food supply, which did not fully account for household production of dairy.^18^ There were 3 initial folate burden ratings that reduced in severity from high to moderate after considering evidence that was excluded from the quantitative burden estimates. For various reasons, 2 vitamin B_12_ and 2 vitamin C burden ratings reduced in severity from high to moderate and 2 niacin burden ratings increased in severity from negligible to low. One iron burden rating and 1 vitamin B_6_ burden rating increased in severity from negligible to low after evidence was considered that was excluded from the quantitative burden estimates.

Six certainty-of-evidence ratings changed from their initial ratings. For various reasons, the vitamin A and vitamin B_12_ certainty ratings for the Maldives increased from low to moderate, the iodine certainty rating for Bangladesh increased from low to moderate, the iodine certainty rating for Nepal increased from moderate to high, the iron certainty rating for Sri Lanka decreased from high to moderate, and the folate certainty rating for Afghanistan decreased from moderate to low.

## FINAL MICRONUTRIENT GAP RATINGS

**Figure 1:**
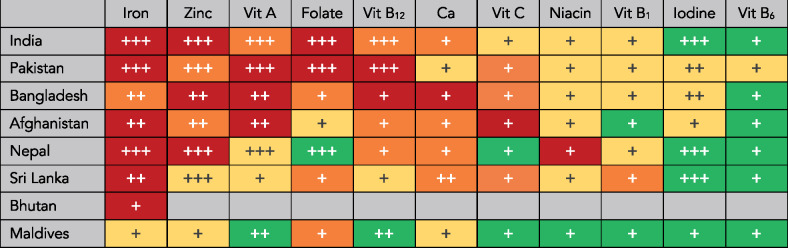
**Gap burden and certainty of evidence ratings for 11 micronutrients among children aged 6–23 months in South Asia.** Nutrient gap burden is signified by color: red (high burden), orange (moderate burden), yellow (low burden), or green (negligible burden). The number of plus (+) signs represents the certainty of evidence: 3 (high certainty), 2 (moderate certainty), or 1 (low certainty). *Abbreviations:* Ca, calcium; Vit, vitamin.

Figure 1 shows the final micronutrient gap burden and certainty-of-evidence ratings for the 11 micronutrients investigated in the 8 countries of South Asia. Gaps in iron are the highest priority micronutrient gaps in South Asia, with estimated high burden gaps in 6 countries, a moderate burden gap in Bangladesh, and a low burden gap only in the Maldives. The iron gap burden in Bangladesh is moderate rather than high, likely because of high iron in groundwater in many areas in Bangladesh.^19^ Other important micronutrient gaps across much of South Asia include zinc, vitamin A, folate, vitamin B_12_, calcium, and, to some extent, vitamin C. Deficiencies in iron, zinc, vitamin A, folate, and vitamin B_12_ can have severe and/or long-term consequences.^6^ Calcium deficiency increases risk of rickets, but the broader health implications of its deficiency in young children are poorly understood.^20^ Vitamin D gaps were not assessed, but available evidence suggests vitamin D may be a high priority gap in India^4^ and particularly Pakistan^21^ and Afghanistan.^22^ There do not appear to be important gaps in iodine or vitamin B_6_ across the region. The Maldives is the only country in the region that does not appear to have important gaps in multiple micronutrients. This is likely because the Maldives has a far higher gross domestic product per capita than any other country in South Asia and high supplies of meat and especially fish.^23^ In Bhutan, the only micronutrient with sufficient qualifying evidence to rate was iron.

The micronutrients with the highest certainty of evidence were iron, zinc, and vitamin A, all of which had moderate or high certainty of evidence in 6 countries. There was a moderate or high certainty of evidence for iodine in 5 countries, folate and vitamin B_12_ in 3 countries each, and calcium in only 1 country. All burden ratings for niacin, thiamine, vitamin C, and vitamin B_6_ were based on low certainty evidence.

New data collection and evidence generation should be prioritized for micronutrients with a moderate or high burden rating and a low certainty rating, as well as micronutrients with no evidence. These include folate, vitamin B_12_, calcium, and vitamin C in Bangladesh; vitamin B_12_, calcium, and vitamin C in Afghanistan; vitamin B_12_, calcium, and niacin in Nepal; folate, vitamin C, and thiamine in Sri Lanka; calcium in India; vitamin C in Pakistan; folate in the Maldives; and all nutrients, but especially iron, in Bhutan.

## NUTRITIOUS FOODS TO FILL IDENTIFIED AND POTENTIAL MICRONUTRIENT GAPS

There are various ways to fill micronutrient gaps in young-child diets, including increasing intake of diverse whole foods, fortified complementary foods, biofortified foods, fortified staples, point-of-use fortification products, supplementation, and improving breastfeeding practices. All these strategies are warranted in South Asia to varying degrees, depending on the local context. Improving intake of minimally processed nutritious whole foods is particularly important, because food influences more than nutrient adequacy (ie, food is more than its nutrients). Minimally processed plant and animal-source foods, including breast milk, contain important beneficial bioactive compounds (eg, polyphenols, fiber, conjugated linoleic acid) that can help regulate the immune system, build a healthy gut microbiome, and ultimately may help reduce risk of overweight or obesity and diet-related noncommunicable diseases in middle childhood, adolescence, and adulthood.^24–28^ Moreover, although overconsumption is not a major concern during the complementary feeding period, increased consumption of highly processed, hyperpalatable, and addictive foods could lead to unhealthy dietary preferences and thus overconsumption and obesity in older childhood and adulthood.^29^^,^^30^ For these reasons, the focus in the following paragraphs is on identifying nutritious whole-food sources of lacking micronutrients.

**Figure 2:**
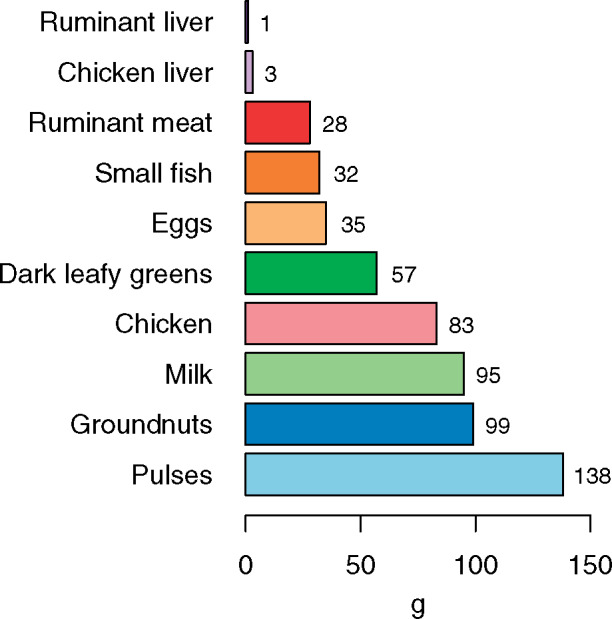
**Portion size needed to achieve an average of 33.3% of requirements for iron, vitamin A, zinc, folate, vitamin B_12_, and calcium from complementary foods in South Asia (each micronutrient capped at 100% of daily requirements).** The proportion of micronutrient requirements from complementary foods was assumed to be 0.98 for iron, 0.87 for zinc, 0.65 for calcium, 0.17 for vitamin A, 0.70 for vitamin B_12_, and 0.60 for folate.^31^ Iron and zinc requirements was adjusted for bioavailability. For iron, it was assumed there was 15% dietary iron bioavailability from animal-source foods and 10% from plant foods; for zinc, it was assumed there was 50% dietary zinc bioavailability from animal-source foods and 30% from legumes, nuts, and seeds^32^

Table 3 provides the micronutrient densities and average share of requirements across 6 micronutrients per 100 g of micronutrient-dense foods that can help address identified and potential micronutrient gaps in the region. These foods may not be available or acceptable in all areas of South Asia or may only be available seasonally. The best whole-food sources of multiple identified or potential micronutrient gaps (iron, zinc, vitamin A, vitamin B_12_, folate, and calcium), as measured by average share of requirements per 100-g portion, are chicken liver, ruminant liver, small fish, eggs, ruminant meat, and dark leafy greens. Small fish are also good sources of vitamin D and long-chain omega-3 fats, which are important for child development and have other health benefits.^34^Figure 2 shows the portion size of each food needed to meet an average of 33.3% of requirements across the same 6 micronutrients. Strikingly, only 1 g of ruminant liver, 3 g of chicken liver, 27 g of ruminant meat, 32 g of small fish, or 35 g of eggs are required to reach this threshold for a child aged 6–23 months, demonstrating the importance of these nutrient-dense animal-source foods in young children’s diets. Larger quantities are required, however, for animal-source foods like chicken and milk to achieve this threshold. Although a moderate-sized portion (57 g) of dark leafy greens can meet the threshold, a much larger portion of pulses (138 g) would be required to achieve the same outcome.

**Table 3.** Micronutrient densities and average share of requirements per 100 g of foods high in priority micronutrients^a^

Food	Iron (mg)	Vit A (RAE)	Calcium (mg)	Zinc (mg)	Folate (DFE)	Vit B_12_ (µg)	Average share of requirements across all 6 micronutrients (%)^b^
Chicken liver	**12.3**	4139	11	4	**569**	19	**84**
Ruminant liver	6.7	**11** **562**	7	5.9	163	**69**	83
Small fish^c^	3	32	**382**	1.9	10	8.9	70
Eggs	1.2	149	50	1.1	44	1.1	59
Ruminant meat	3.5	0	11	**6.1**	7	2.7	48
Dark leafy greens	2.7	367	151	0.6	54	0	46
Chicken	1.2	46	14	1.9	5	0.3	42
Milk	0	46	113	0.4	5	0.5	37
Groundnuts	1.3	0	57	2.3	86	0	34
Fresh peas	2.1	54	43	0.3	42	0	33
Mango	0.5	152	13	0.1	33	0	26
Pulses	2.6	1	34	1.3	45	0	24
Carrot/pumpkin	0.5	570	23	0.2	12	0	22
Okra	0.3	14	77	0.4	46	0	21

a All foods are in the form typically consumed. Values, except small fish, are from Ryckman et al^15^ in this issue of *Nutrition Reviews*. Bold numbers indicate the highest density of the specified micronutrient or average share of requirements. *Abbreviations:* DFE, dietary folate equivalent; RAE, retinal activity equivalent.

b Average share of requirements for iron, zinc, vitamin A, vitamin B_12_, folate, and calcium per 100 g, assuming requirements from complementary foods for children aged 6–23 months (each micronutrient capped at 100% of daily requirements). The proportion of micronutrient requirements from complementary foods were assumed to be 0.98 for iron, 0.87 for zinc, 0.65 for calcium, 0.17 for vitamin A, 0.70 for vitamin B_12_, and 0.60 for folate.^31^ Iron and zinc requirements were adjusted for bioavailability. For iron, we assumed 15% dietary iron bioavailability for animal-source foods and 10% for plant foods; for zinc we assumed 50% dietary zinc bioavailability for animal-source foods and 30% for legumes, nuts, and seeds.^32^

c Nutrient values from canned sardines in the US Department of Agriculture food composition data.^33^

According to Food and Agriculture Organization Food Balance Sheets, South Asia has the lowest per capita availability of meat (19 g/day) of any region globally, and India the lowest of any country (10 g/day).^23^ However, given young children’s low overall food intake, social and behavioral change communication campaigns and counselling on child diets could encourage the increased allocation of existing supplies of chicken liver, ruminant liver, and ruminant meat for children aged 6–23 months and provide meaningful intake of bioavailable nutrients. Small fish are only widely available in the Maldives, Sri Lanka, and Bangladesh; in other South Asian countries, they may only be available in specific geographic areas (eg, fresh small fish in areas near water or tinned small fish in select cities).^23^ Fewer than 10 g of eggs are available per capita in South Asia,^23^ which indicates a need for preferential allocation of existing supplies to young children and/or increased production. The availability of dark leafy greens is less clear, but they are likely available at least in moderate quantities in most areas in South Asia.^15^ For differences in household consumption of common whole-food sources among India, Bangladesh, and Pakistan, refer to Ryckman et al.^15^

## CONCLUSION

Unsurprisingly, there was heterogeneity in micronutrient gap burdens and certainty ratings across countries and micronutrients in South Asia. Although micronutrient gaps varied across countries, most countries had identified or potential gaps in multiple micronutrients. Diets were apparently inadequate in micronutrients, especially iron, zinc, vitamin A, folate, vitamin B_12_, calcium, and vitamin C. Previous literature on regional patterns of micronutrient gaps in South Asia is limited. In accordance with our findings in the present study, a review of iron, iodine, vitamin A, and zinc deficiencies in South Asia found that there was a low burden of iodine deficiency (largely a result of widespread salt iodization in the region) but a high burden of iron, vitamin A, and zinc deficiencies.^3^ Animal-source foods, particularly liver, small fish, eggs, and ruminant meat, are the best sources of the highest priority micronutrients but likely have major obstacles to increased consumption, including availability,^18^ affordability,^15^ access, knowledge, and cultural preferences.^35^ Dark leafy greens are also a good source of multiple high-priority micronutrients. Special attention should be given to including these diverse sources of micronutrients in young children’s diets.

Evidence on micronutrient gaps was most robust in India, Pakistan, and Nepal, because of their recent national micronutrient surveys. In accordance with a CONGA on Eastern and Southern Africa,^17^ across South Asia there was limited biochemical evidence on folate, vitamin B_12_, and especially calcium deficiencies, and no evidence for vitamin C, niacin, thiamine, or vitamin B_6_ deficiencies. Among these, investment is warranted in collecting biomarkers on folate, vitamin B_12_, calcium, and vitamin C status where recent evidence does not exist, given that low-certainty evidence suggests there are potential gaps in these micronutrients in several South Asian countries. Although vitamin D gaps were not assessed, deficiency is common in at least parts of South Asia,^4^^,^^21^^,^^22^ which suggests vitamin D biomarkers are important to collect across the region. Biochemical data should be collected at the national level at least every 10 years, and ideally more frequently, to monitor programs, track progress, and inform policies and programs. Importantly, there was very little evidence from studies assessing micronutrient adequacy of individual or household diets, a clear gap in collection and/or use of dietary intake and household consumption data in the region. Other than for iron, there was not enough evidence in Bhutan to assess micronutrient gaps, highlighting the urgent need to collect and analyze biomarker, dietary, and household consumption data in the country. Overall, however, there was much more robust evidence on micronutrient gaps in South Asia than was identified by a related CONGA on Eastern and Southern Africa.^17^

Assessing micronutrient gaps in young-child diets across South Asia using the CONGA method allowed for a critical assessment of the relevant evidence from different types of data sources that are not usually synthesized (ie, biochemical, nutrient intake and availability, and nutrient-informative food-group intake). This comprehensive assessment addresses this knowledge gap and provides a more complete understanding of micronutrient gaps in each country than would have been possible if relying on only 1 type of data or a subset of the evidence base. Moreover, evidence sources, even robust sources, often have conflicting findings. Therefore, the use of multiple sources reduced the likelihood of erroneous conclusions. Furthermore, assessing the certainty of the micronutrient gap ratings provided transparency about the evidence behind them, which will help limit inappropriate use of evidence or overconfidence in findings that are not robust, representative, validated, or recent. Finally, because CONGA is a standardized process,^6^ it enabled valid comparisons across countries.

This assessment has important limitations. A systematic search was not conducted to identify evidence sources for inclusion in the CONGA. Although it is unlikely that robust, large-scale evidence sources were not identified, it is likely that some qualifying evidence was not. Thus, it is unclear how many additional resources would have been included in a systematic review and the extent to which their inclusion would have influenced ratings. Also, although the CONGA method was designed to be standardized and minimize bias, it has built-in flexibility to allow reviewers to override automated ratings on the basis of their qualitative assessment of the total evidence base, including data points excluded from the automated rating process. As a result, the final ratings, to some extent, are a reflection of the subjective perspective of experts who reviewed the evidence. The impact of this bias was minimized by having 2 experts make the initial ratings and revisions jointly and multiple subject-matter and local experts review and validate the final ratings. Notably, although the CONGA method can be applied subnationally, we did not assess in this analysis subnational differences in nutrient gaps (which can vary considerably within countries) or foods to fill these gaps. In addition, there may be other nutrient gaps that we did not identify in this analysis because there was limited robust evidence for several micronutrients. Finally, differences in ratings across countries may be due, in part, to differences in the date of data collection across countries. Additional limitations of the CONGA method are specified elsewhere.^6^

The findings from this CONGA are essential for addressing micronutrient malnutrition and inadequate diets among young children in South Asia. They provide a clear understanding of the specific issues and limitations in data on micronutrient gaps and evidence across the region. The numerous causes of these important micronutrient gaps should be further studied and addressed through a food-systems approach that identifies and removes the largest barriers to adequate intakes, including availability, accessibility, affordability, cultural acceptability, desirability, convenience, knowledge, and feeding practices.^36^ For example, only 57% of the population in South Asia can afford a healthy diet^37^; in many areas, animal-source foods are not widely available^23^; and cultural preferences make it challenging to ensure young-child diets are adequately nutrient dense.^35^ Poor dietary diversity and quality are modifiable risk factors underlying child malnutrition in South Asia. Addressing all context-specific barriers contributing to micronutrient gaps will go a long way in accelerating reductions in child malnutrition, which affects millions of children, their futures, and society at large across South Asia and beyond.

Policy and programmatic interventions to address micronutrient gaps should be guided by a systematic analysis of context-specific determinants and by drivers influencing access to and consumption of nutritious and safe whole-food sources, identified in this study, that can fill critical micronutrient gaps during the complementary feeding period. Finally, a multisystem response involving the health, food, water and sanitation, and social protection systems is warranted in countries in the region to collectively create the necessary conditions for micronutrient-adequate diets and adequate feeding practices.

## Supplementary Material

nuaa144_Supplementary_Data

## Supporting Information

*Table S1* **Search terms used in the literature review**

*Supplementary Material:* **References that qualified for inclusion in the CONGA in South Asia**

*Table S2* **Spreadsheet of nutrient gap burden and certainty-of-evidence ratings in the 8 countries included in this study** 
