# Supplementary material for: Micronutrient gaps during the complementary feeding period in South Asia: A Comprehensive Nutrient Gap Assessment
Source: Nutr Rev. 2021 Mar 8;79(Suppl 1):26–34. doi: 10.1093/nutrit/nuaa144 (PMC7947968; doi:10.1093/nutrit/nuaa144)
Supplement: nuaa144_Supplementary_Data [file nuaa144_supplementary_data.zip › Supplemental Material - References.docx]

**References that qualified for inclusion in the CONGA in South Asia**

Abeywickrama HM, Koyama Y, Uchiyama M, et al. Micronutrient Status in Sri Lanka: A Review. *Nutrients.* 2018;10(11).

Aga Khan University, Ministry of Health, UNICEF. *Project Report - National Micronutrients Survey 2007.* 2010.

Atwood SJ, Nagpal S, Mbuya NV, Lavioletted L. *Nutrition Situation in Bhutan: Situational Analysis and Policy Recommendations.* The International Bank for Reconstruction and Development / The World Bank;2014.

Beal T, Massiot E, Arsenault JE, Smith MR, Hijmans RJ. Global trends in dietary micronutrient supplies and estimated prevalence of inadequate intakes. *PLoS One.* 2017;12(4):e0175554.

Campbell RK, Aguayo VM, Kang Y, et al. Infant and young child feeding practices and nutritional status in Bhutan. *Matern Child Nutr.* 2018;14 Suppl 4:e12762.

Campbell RK, Aguayo VM, Kang Y, et al. Epidemiology of anaemia in children, adolescent girls, and women in Bhutan. *Matern Child Nutr.* 2018;14 Suppl 4:e12740.

Central Statistics Organization/Afghanistan, Ministry of Public Health/Afghanistan, ICF. *Afghanistan Demographic and Health Survey 2015.* Kabul, Afghanistan: Central Statistics Organization;2017.

DCS, MoHNIM. *Sri Lanka Demographic and Health Survey 2016.* Sri Lanka: Department of Census and Statistics (DCS) and Ministry of Health, Nutrition and Indigenous Medicine;2017.

Dzed L, Dorji T, Pelzom D, Dhakal G, Yangchen P, Wangmo K. Status of Thiamin deficiency in boarding school children from seven districts in Bhutan with previous history of peripheral neuropathy outbreaks: a cohort study. *Bhutan Health Journal.* 2015(1):49-56.

Food Security and Agriculture Cluster. *Afghanistan Emergency Food Security Assessment: August-September 2018.* Food Security and Agriculture Cluster (FSAC); Ministry of Agriculture Irrigation and Livestock (MAIL);2018.

GHNC. *SAARC Development Goals: Mid-term Review Report.* Thimphu: Gross National Happiness Commission;2011.

Government of Pakistan Planning and Development Division. *National Nutrition Survey 2011.* 2011.

icddr b, UNICEF, GAIN, IPHN. *National Micronutrients Status Survey 2011-12.* International Centre for Diarrhoeal Disease Research Bangaldesh, UNICEF Bangladesh, Global Alliance for Improved, Nutrition and the Institute of Public Health and Nutrition 2013.

ICF. The DHS Program STATcompiler. http://www.statcompiler.com. Accessed January 10, 2020.

International Institute for Population Sciences, ICF. *India National Family Health Survey NFHS-4 2015-16.* Mumbai, India: IIPS and ICF;2017.

International Institute for Population Sciences, Macro International. *India National Family Health Survey (NFHS-3) 2005-06.* Mumbai, India: IIPS and Macro International;2007.

Iodine Global Network. Across India, women are iodine sufficient. In. *IDD Newsletter;*November 2015.

Jayatissa RLN, Wickramasinghe WD, Piyasena C. *Food consumption patterns in Sri Lanka.* Colombo, Sri Lanka: Hector Kobbekaduwa Agrarian Research and Training Institute;2014.

Jayawardena R, Byrne NM, Soares MJ, Katulanda P, Hills AP. Food consumption of Sri Lankan adults: an appraisal of serving characteristics. *Public Health Nutr.* 2013;16(4):653-658.

KIT Royal Tropical Institute. *Afghanistan Health Survey 2018.* 2018.

Knowles JM, Garrett GS, Gorstein J, et al. Household Coverage with Adequately Iodized Salt Varies Greatly between Countries and by Residence Type and Socioeconomic Status within Countries: Results from 10 National Coverage Surveys. *J Nutr.* 2017;147(5):1004S-1014S.

Mark HE, Houghton LA, Gibson RS, Monterrosa E, Kraemer K. Estimating dietary micronutrient supply and the prevalence of inadequate intakes from national Food Balance Sheets in the South Asia regiona. *Asia Pacific journal of clinical nutrition.* 2016;25(2):368-376.

Medical Research Institute, UNICEF, WFP. *National Nutrition and Micronutrient Survey of Pregnant Women in Sri Lanka 2015.* 2017.

Medical Research Institute, UNICEF, WFP, Ministry of Health. *Iodine Deficiency Status in Sri Lanka 2016 Fourth National Survey.* 2016.

Ministry of Health. *2015 National Nutrition Survey.* Thimphu, Bhutan: Nutrition Program, Department of Public Health, Ministry of Health. 2015.

Ministry of Health, ICF. *Maldives Demographic and Health Survey 2016-17.* Malé, Maldives, and Rockville, Maryland, USA: MoH and ICF. 2018.

Ministry of Health, UNICEF. *National Nutrition and Micronutrient Survey 2012 Part II: Iron, Zinc and Calcium Deficiency Among Children Aged 6–59 Months.* 2012.

Ministry of Health and Family Maldives, ICF Macro. *Maldives Demographic and Health Survey 2009.* Calverton, Maryland, USA: MOHF and ICF Macro;2010.

Ministry of Health and Family Welfare, Government of India, UNICEF, Population Council. *Comprehensive National Nutrition Survey 2016-2018.* New Delhi2019.

Ministry of Health and Population, New ERA, UNICEF, EU, USAID, CDC. *Nepal National Micronutrient Status Survey, 2016.* Kathmandu, Nepal: Ministry of Health and Population, Nepal. 2018.

Ministry of Health and Population Nepal, New ERA, ICF International. *Nepal Demographic and Health Survey 2011.* Kathmandu, Nepal: MOHP/Nepal, New ERA, and ICF International. 2012.

Ministry of Health and Population Nepal, New ERA, Macro International. *Nepal Demographic And Health Survey 2006.* Kathmandu, Nepal: MOHP/Nepal, New ERA/Nepal, and Macro International. 2007.

Ministry of Health Nepal, New ERA, ICF. *Nepal Demographic and Health Survey 2016.* Kathmandu, Nepal: MOH/Nepal, New ERA, and ICF. 2017.

Ministry of National Health Services Regulations and Coordination, UNICEF. *Cost of the Diet Analysis Report in 12 Districts, 17 Livelihood Zones: Pakistan.* UNICEF Pakistan and Ministry of National Health Services, Regulations and Coordination, Government of Pakistan. 2018.

Ministry of National Health Services Regulations and Coordination, UNICEF. *Optifood Analysis Report Pakistan.* UNICEF Pakistan and Ministry of National Health Services, Regulations and Coordination, Government of Pakistan.2018.

Ministry of Planning Development & Reform, WFP. *Fill the Nutrient Gap Report: Pakistan.* Islamabad. 2018.

Ministry of Public Health, UNICEF. *National Nutrition Survey Afghanistan 2013.* 2013.

Ministry of Women and Child Development, UNICEF. *Rapid Survey on Children (RSOC) 2013/14 National Report.*

National Institute of Population Research Training, Mitra Associates, ICF International. *Bangladesh Demographic and Health Survey 2011.* Dhaka, Bangladesh: NIPORT, Mitra and Associates, and ICF International. 2013.

National Institute of Population Research Training, Mitra Associates, ICF International. *Bangladesh Demographic and Health Survey 2014.* Dhaka, Bangladesh: NIPORT, Mitra and Associates, and ICF International. 2016.

National Institute of Population Studies, ICF International. *Pakistan Demographic and Health Survey 2012-13.* Islamabad, Pakistan: NIPS/Pakistan and ICF International. 2013.

National Institute of Population Studies, Macro International. *Pakistan Demographic and Health Survey 2006-07.* Islamabad, Pakistan: NIPS/Pakistan and Macro International. 2008.

National Nutrition Monitoring Bureau. *Diet and nutritional status of rural population, prevalence of hypertension & diabetes among adults and infant & young child feeding practices: Report of third repeat survey.* Hyderabad: Indian Council of Medical Research. 2012.

National Nutrition Monitoring Bureau. *Diet and nutritional status of urban population in India and prevalence of obesity, hypertension, diabetes and hyperlipidemia in urban men and women: NNMB brief report on urban nutrition.* Hyderabad: Indian Council of Medical Research. 2017.

National Nutrition Monitoring Bureau. *Prevalence of vitamin A deficiency among preschool children in rural areas.* Hyderabad: Indian Council of Medical Research. 2006.

Nutrition Wing Ministry of National Health Services Regulations and Coordination. *National Nutrition Survey 2018: Key Findings Report.* Government of Pakistan. 2019.

Weerahewa J, Gedara P, Wijetunga C. Nutrition Transition in Sri Lanka: A Diagnosis. *Ann Nutr Food Sci.* 2018;2(2).

WHO. *Global prevalence of vitamin A deficiency in populations at risk 1995–2005. WHO Global Database on Vitamin A Deficiency.* Geneva: World Health Organization;2009.

Wieser S, Brunner B, Tzogiou C, et al. Societal Costs of Micronutrient Deficiencies in 6- to 59-month-old Children in Pakistan. *Food Nutr Bull.* 2017;38(4):485-500.

Wirth JP, Petry N, Tanumihardjo SA, et al. Vitamin A Supplementation Programs and Country-Level Evidence of Vitamin A Deficiency. *Nutrients.* 2017;9(3).
