# Supplementary material for: Micronutrient gaps during the complementary feeding period in South Asia: A Comprehensive Nutrient Gap Assessment
Source: Nutr Rev. 2021 Mar 8;79(Suppl 1):26–34. doi: 10.1093/nutrit/nuaa144 (PMC7947968; doi:10.1093/nutrit/nuaa144)
Supplement: nuaa144_Supplementary_Data [file nuaa144_supplementary_data.zip › Table S1.docx]

**Table S1. Search terms used in the literature review.**

| Evidence type  Search engine: Google and Google scholar | Specific report types or grey literature  Search engine: Google |
| --- | --- |
| Infant and young child feeding  Nutrient availability  Nutrient gap + children  Nutrient intake + children  Dietary quality + children  Dietary intake + children  Food group + children  Food consumption + children  Child diet  Complementary foods  Complementary feeding  Biochemical status + children  Micronutrient deficiency + children | Micronutrient survey  Food consumption survey  Nutrition survey  Household income and expenditure survey  Optifood  Cost of the diet  Fill the Nutrient Gap |
| *Search terms were included paired both with region (Eastern and Southern Africa), country names and nutrients of interest | |
